# Tooth Whitening with Hydroxyapatite: A Systematic Review

**DOI:** 10.3390/dj11020050

**Published:** 2023-02-12

**Authors:** Hardy Limeback, Frederic Meyer, Joachim Enax

**Affiliations:** 1 Faculty of Dentistry, University of Toronto, Toronto, ON M5G 1G6, Canada; 2 Dr. Kurt Wolff GmbH & Co. KG, Research Department, Johanneswerkstr. 34-36, 33611 Bielefeld, Germany

**Keywords:** hydroxyapatite, systematic review, in vitro, in vivo, clinical trial, RCT, teeth, toothpaste, whitening

## Abstract

A steadily increasing public demand for whiter teeth has resulted in the development of new oral care products for home use. Hydroxyapatite (HAP) is a new ingredient to whiten teeth. This systematic review focuses on the evidence of whether HAP can effectively whiten teeth. A systematic search using the PICO approach and PRISMA guidelines was conducted using PubMed, Scopus, Web of Science, SciFinder, and Google Scholar as databases. All study designs (in vitro, in vivo) and publications in foreign language studies were included. Of the 279 study titles that the searches produced, 17 studies met the inclusion criteria. A new “Quality Assessment Tool For In Vitro Studies” (the QUIN Tool) was used to determine the risk of bias of the 13 studies conducted in vitro. Moreover, 12 out of 13 studies had a low risk of bias. The in vivo studies were assigned Cochrane-based GRADE scores. The results in vitro and in vivo were consistent in the direction of showing a statistically significant whitening of enamel. The evidence from in vitro studies is rated overall as having a low risk of bias. The evidence from in vivo clinical trials is supported by modest clinical evidence based on six preliminary clinical trials. It can be concluded that the regular use of hydroxyapatite-containing oral care products effectively whitens teeth, but more clinical trials are required to support the preliminary in vivo evidence.

## 1. Introduction

The primary goal of modern oral care products is to treat or prevent dental disease, such as caries and periodontal disease, and to maintain good oral health. For many reasons, there has been a decline in dental disease in developed countries, resulting in people retaining their teeth into old age [1]. People with disease-free dentitions value the cosmetic appearance of their teeth, and whiter teeth suggest good dental health, improving confidence and self-esteem, as well as social acceptance [2], and even improved employment prospects [3]. People today seek out oral care products that are believed to make teeth “whiter, brighter, and healthier looking”. This is partially driven by the white smiles of celebrity role models or magazine models [4], by the successful advertising of new “whitening” oral care products from toothpaste manufacturers [5], and by the dental profession offering patients new ways to whiten their teeth [6].

With age, as the enamel becomes thin, teeth naturally lose their luster and whiteness, and as pulp chambers decrease in size, the underlying yellow dentin becomes more visible [7]. Intrinsic staining from exposure to various drugs, including fluoride, before and after tooth development can significantly alter the color of teeth [8]. Stannous fluoride added to the toothpaste can produce noticeable staining [9,10]; however, this problem was minimized with stabilization of the ions [9,11]. Products containing chlorhexidine, such as mouthwashes to treat periodontitis, cause significant surface staining, in addition to some undesirable side effects [12]. Inadequate oral hygiene with tooth brushing and toothpaste will result in more than usual acquired extrinsic staining from everyday exposure to food components, such as tannin from tea drinking, coffee, and wine stains, as well as tar and nicotine stains from tobacco use [13]. Even the buildup of plaque and calculus, both of which can darken and cover the clean enamel surfaces with time, in the absence of regular professional cleaning, is quite noticeable [14,15].

Many approaches and techniques have been used to whiten teeth, both in the dental office and at home [16]. In the dental office, the dentist or dental hygienist typically removes dental calculus with cavitation or hand instruments, followed by surface stain removal using abrasives [17]. If patients want whiter teeth than what would be normal for their age, they typically choose to have professional whitening treatments performed, which almost always involve increasing concentrations of carbamide peroxide with or without light activation [18]. However, the treatments with high concentrations of peroxide lead to many undesirable side effects [19]. Despite this, vital bleaching has become a very common procedure in dental offices and in non-dental settings. Moreover, at home, patients want to use oral care products that will maintain the new whiteness of their teeth or whiten their teeth if they choose not to have professional treatment. There are many options for whitening teeth at home. Vital bleaching kits are available over-the-counter, which utilize gel strips with peroxide or home trays into which peroxide gels are placed [6]. However, the most common home care approaches to tooth whitening are the repeated use of whitening toothpastes and mouthwashes. Numerous different nanoparticles have been developed for the purpose of producing toothpastes and mouth rinses that effectively whiten teeth [20]. Most toothpastes produce their whitening effect through the abrasive additives that remove stains [13] or through oxidation with peroxide which chemically changes dental proteins [21]; however, some colorants, such as covarine deposited on the tooth surface, provide immediate perception of whiter teeth [22,23]. Unfortunately, some whitening toothpastes are very abrasive and can lead to dental wear [24]. An optimal balance between abrasiveness and whitening effect is difficult to attain when a toothpaste’s main mechanism of whitening is through the abrasives they contain. In addition, toothpastes containing peroxide, which is meant for daily use, may have additional deleterious effects on the enamel structure [25]. Vital soft tissues in the oral cavity [26], and even within the tooth [27], may be negatively affected.

Therefore, there is a need for the use of safer whitening agents in toothpastes. The ingredients should have low abrasivity, but at the same time, significantly whiten teeth. One of these ingredients is hydroxyapatite. Biomimetic hydroxyapatite particles are white opaque particles providing immediate repair of surface enamel defects. In the long-term, this active ingredient has been shown to remineralize tooth surfaces, reducing caries [28,29] and dentin hypersensitivity [30,31]. The deposition of hydroxyapatite particles on the enamel surface not only masks the yellow appearance of teeth, but also effectively removes extrinsic stains and plaque, as demonstrated in vitro and in some clinical trials [16,32]. The addition of hydroxyapatite into the toothpaste has been extensively studied and approved for sale in many countries, originally starting in Japan [20], then in Europe (e.g., Germany, Italy), and now in Canada [33].

In this review, we performed a systematic literature search and quality assessment of in vitro and in vivo evidences, which show that hydroxyapatite-containing oral care formulations whiten teeth. We found sufficient evidence to conclude this, and herein provide a discussion of the modes of action of how hydroxyapatite safely whitens teeth.

## 2. Materials and Methods

A PICO framework was used to guide the search. The following question was posed “Do oral care products containing hydroxyapatite effectively whiten teeth”? The target population (P) was dental tissues (in vitro or in vivo); the intervention (I) was using hydroxyapatite in a synthetic slurry, or in a commercial toothpaste or mouthwash; the comparison (C) was untreated tissue or placebo, or positive controls (other toothpastes or mouthwashes); and the outcome (O) was a change in the color or brightness of the treated tissue or dentition.

Although this systematic review was not registered with PROSPERO, our protocol followed the PRISMA guidelines (see PRISMA-S checklist in the supplement) [34]. The literature was simultaneously searched by one author (H.L.) and separately by two other authors (F.M. and J.E.). H.L. used the University of Toronto databases PubMed (Medline), Scopus, Web of Science, as well as Google Scholar, from inception to 31 October 2022. F.M. and J.E. independently searched PubMed, SciFinder, and Google Scholar. The search terms were “hydroxyapatite” and “white” and/or “whitening” and “dental” and/or “teeth” and “toothpaste” and/or “dentifrice” and/or “mouthwash”. Duplicates and those publications whose titles clearly indicated no reporting of oral issues of dental whitening were eliminated. Then, abstracts were read to further eliminate publications unrelated to the goals of the review.

Inclusion and exclusion criteria: Review articles, abstracts, book chapters, and studies on live animals were not included. All included studies were in vitro studies that examined animal or human teeth or sections of teeth as well as in vivo studies that involved humans (clinical studies, randomized controlled trials, or RCTs). Moreover, studies published in foreign languages were included.

Microsoft Excel spreadsheets were used to generate publication lists manually by downloading the particulars of each publication (authors, title, journal, abstract, key words), or “cvs” files were automatically generated by the databases, such as Scopus. Duplicates were manually removed once the studies were listed alphabetically. All authors collaborated and reached a consensus on which studies to include. Once the decision was made regarding which publications to include, the electronic version of each publication was retrieved in full and read in detail. Studies published in a foreign language were translated using Google Translate. H.L. was responsible for the assignment of the qualitative ratings, and the co-authors were consulted for agreement on the gradings. The quality of the in vitro studies was assessed according to a new risk of bias assessment tool for in vitro dental studies [35]. The quality of the in vivo studies was assessed at the outcome level based on the GRADE approach [36]. The ratings of “Very Low, Low, Moderate” were also based on whether there were deficiencies in the study design, and not only on the risk of bias. A qualitative meta-analysis was not possible given the heterogeneity of the studies.

## 3. Results

**Figure 1:**
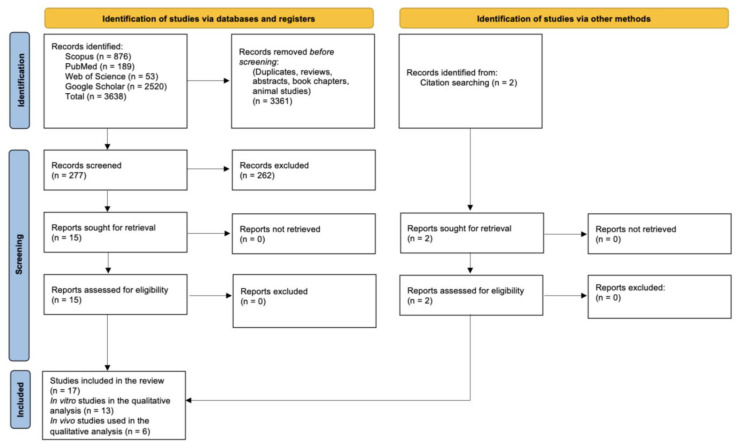
PRISMA flow chart of literature search results (please note that two publications reported separate in vitro and in vivo studies).

In total, 279 titles with abstracts were initially retrieved and screened. After independent searches, consultation, and consensus, the authors’ collaboration resulted in the selection of 17 studies that met the inclusion criteria (Figure 1). The majority of the publications were reviews, or studies unrelated to the whitening of teeth by HAP, or did not meet the PICO requirements of our review.

Eleven publications reported in vitro studies, four were in vivo studies, and two reported both in vitro and in vivo studies. The majority of the studies were conducted in Germany. Summaries of the details of the studies are presented in Table 1, Table 2 and Table 3.

The studies conducted in vitro had mixed designs. Some used whole teeth, others used enamel blocks. Eight experiments used bovine incisors and four used extracted human teeth. Color changes were usually measured by spectrophotometry or shade guides. Only one of the 13 in vitro studies conducted had medium risk of bias (<70% QUIN Tool score). The remaining had low risk of bias (>70% QUIN Tool score). Table 2 contains the QUIN Tool scores for the 13 studies conducted in vitro. These studies all consistently showed improved whitening of HAP toothpaste in vitro.

The results of the systematic search for in vivo evidence resulted in six studies [42,49,50,51,52,53] ranging in quality from very low to moderate (Table 3). Two studies were claimed to be double blinded, randomized clinical trials (RCTs) [52,53], but details were not provided. Studies were ranked “very low” or “low” if they had small sample sizes, did not conduct the clinical trials under single or double-blinded conditions, did not randomize the selection of subjects (or did not report how they conducted the trial to achieve randomization or blinding), or had imprecise measurement techniques of whitening (e.g., questionnaires) or used inappropriate HAP concentrations incapable of detecting whitening effects. Only one study [42] was rated “moderate” since it had a large enough sample size, was placebo controlled, and had the best color change measuring technique. Since the study was not an RCT, it failed to reach the “high” rating. A meta-analysis could not be conducted due to the large heterogeneity between studies. This was due to varying clinical conditions and different ingredient contents.

**Table 3:** Overview of the in vivo hydroxyapatite (HAP) studies in the field of tooth whitening (sorted by publication year, in descending order).

Study (Country) [Reference]	Type of Study	Clinical Study Details	Tested Materials and Products	Conclusion of the Publication Authors	GRADE Assignment of HAP Whitening Effect Conclusion of the Authors of This Review
Steinert, Kuchenbecker et al., 2020 (Germany) [50]	in vivo	Twenty-five subjects; subjective change in tooth color after 4 weeks of use; -questionnaire; -no control, no blinding.	HAP gel.	“In conclusion, microcrystalline hydroxyapatite is a promising whitening agent for oral care formulations and represents a biomimetic alternative to other whitening agents for daily dental care”.	LOW subjective HAP-whitening effect reported.
Steinert, Zwanzig et al., 2020 (Germany) [51]	in vivo	Forty-six subjects; -28 days of trial brushing at home; -VAS scale for color change; -no control (before and after design); -questionnaire results.	Toothpaste with 20% biomimetic zinc HAP.	“Additionally, patients reported smoother and whiter teeth after using the HAP toothpaste”.	LOW HAP-whitening effect confirmed.
Bommer et al., 2018 (Switzerland, Germany) [42]	in vivo	-Forty subjects; -1 month; -no treatment and placebo controls; -whitening index was used to measure the effect.	Mixture of self-assembling peptide matrix and HAP.	“The combination of SAPM+HA particles caused optical whitening based on diffuse reflection by the HA particles on the tooth surface. The whitening effect and its magnitude observed in vitro were also seen in vivo”.	MODERATE HAP-whitening effect confirmed.
Woo et al., 2014 (Korea) [52]	in vivo	-Eighty-five subjects; -3 months of trial; -VAS scale of whitening; -double-blinded, randomized.	Toothpastes containing 0.75% hydrogen peroxide, 0.25% HAP, and placebo.	“The hydrogen peroxide-containing toothpaste caused significant lightening of tooth coloration, (mopre) than the hydroxyapatite and placebo toothpastes”.	LOW -only a very small amount of HAP was used (0.25% HAP), other studies tested concentrations up to 20-30% HAP; -a higher concentration of hydrogen peroxide (0.75%) was used as the control than is allowed in cosmetic toothpastes in the EU (0.1%).
Raoufi and Birkhed 2010 (Sweden) [53]	in vivo	-One-hundred and fifty subjects; -12 weeks of study; -Vita Easy shade test; -double-blinded, randomized.	Toothpastes containing HAP, calcium peroxide, and placebo.	“The toothpaste containing hydroxyapatite or calcium peroxide did not produce any reduction in tooth staining compared with a placebo fluoride toothpaste”.	LOW -only a very small amount of HAP was used (0.1% HAP or less; no exact amount was given); other studies tested concentrations up to 20–30% HAP; -interestingly, even the peroxide group was not superior to a control toothpaste.
Niwa et al., 2001 (Japan) [49]	in vivo	-Twelve subjects; -4 weeks of trial; -colorimeter with fiberscope to measure brightness and whiteness; -five volunteers in the 3% HAP group, seven volunteers in the 15% HAP group (no details of blinding, randomization).	Toothpastes containing 3% and 15% HAP were compared to the placebo (no HAP).	“It is concluded that toothpaste containing hydroxyapatite are effective at whitening tooth and that whitening was not due to their polishing effect on tooth surface”.	LOW HAP-whitening effect confirmed (15% HAP more efficient that 3% HAP).

## 4. Discussion

Although an improvement in the appearance of teeth (“whiter and brighter”) has been the goal of toothpaste manufacturers for many decades [54], the literature on non-peroxide tooth whitening is limited [16]. Well-conducted clinical trials are lacking due to the complexity of toothpaste formulations. However, some comparisons of commercial products have been carried out. For example, stain removal of fluoride toothpastes depends on their abrasiveness as measured by the relative dentin abrasivity (RDA) value [24,55,56].

### 4.1. Abrasivity

In the review by Epple et al. 2019 [16], a comparison was made between the abrasivity of various toothpaste ingredients, where perlite and alumina were considered as hard abrasive additives; sodium bicarbonate, sodium carbonate, and brushite (DCPD or dicalcium phosphate dihydrate) were considered as soft abrasive additives; and hydroxyapatite along with hydrated silica and calcium pyrophosphate were considered to have medium abrasivity. Removal of dental stain and plaque requires a balance between effectiveness of abrasion and protection against dental wear. Dental wear can occur with very abrasive toothpastes, which are administered with aggressive and frequent brushing. Clearly, where the loss of tooth structure occurs, the toothpaste formulation needs to be improved. The American Dental Association issued a statement that “All dentifrices with the Seal of Acceptance must have an RDA of 250 or less” [57]. This upper limit, however, allows nearly all toothpastes on the market to claim that they are not abrasive, which may be misleading. The “roughness” of the abrasive agent must also be considered [58]. The fluoride agent in the toothpaste might provide some protection against dental wear [59], but toothpastes with low RDA values will also demonstrate dentin wear depending on the various conditions, such as toothbrush bristle diameter [60,61]. Therefore, dental wear from toothpaste use is multifactorial, and using RDA alone does not predict whether a certain toothpaste product is safe to use in terms of abrasivity. The conclusion of the Niwa et al. study [49] was that tooth whitening was not achieved through any abrasiveness that a higher concentration of HAP might have produced.

### 4.2. Peroxide Side Effects

The oxidation side effects of using high concentrations of peroxide to whiten teeth with in-office and home vital bleaching techniques have been reported; however, even low concentrations of peroxides, such as those used in toothpastes in Canada and the US, may be harmful [6,16,19,20,27]. In-office and home bleaching with peroxide results in increased dentin hypersensitivity, but this is not made worse with the application of intense light (“power bleaching”) [18]. Tooth sensitivity from vital dental bleaching can be partially alleviated when hydroxyapatite is applied before the peroxide application [62,63]. When HAP-containing toothpastes are used after vital bleaching, they effectively reduce dentin hypersensitivity [31] and continue to maintain whiteness [44].

### 4.3. Mechanism of Whitening with HAP Toothpaste

**Figure 2:**
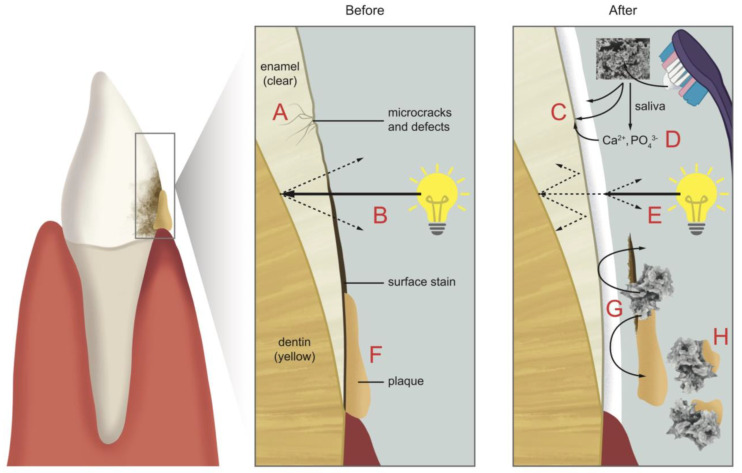
Proposed mechanisms involved in hydroxyapatite tooth whitening. The incisor showing the plaque and stain (**left**) is illustrated in the cross-section before (**middle**) and after (**right**) the administration of HAP-containing toothpaste. Before: (A) Microcracks and defects in the enamel lower the luster of enamel; (B) healthy enamel is basically clear, and the underlying dentin which is yellow, will result in a tooth that appears off-white or yellow; (F) deposits of stain and plaque on the tooth surfaces are yellow or dark colored, giving teeth a yellow appearance. After: (C) Applied hydroxyapatite from the toothpaste adheres to the enamel (a drawing of HAP clusters from a typical SEM is shown), repairing microcracks and surface defects; (D) some hydroxyapatite molecules dissociate into excess calcium and phosphate ions, which also diffuse into the microcracks and onto the surface enamel to contribute to the remineralization of the surface enamel; (E) long-term use of hydroxyapatite toothpaste results in a white opaque hydroxyapatite mineral layer that blocks incoming light and reflects back white light. Light that manages to reach the dentin and reflects back through the enamel is blocked by the white opaque hydroxyapatite layer. The net result is a whitening effect of the tooth since the dentin is less visible; (G) hydroxyapatite from the toothpaste is mildly abrasive and effective in removing stain and plaque, resulting in whiter teeth; (H) hydroxyapatite from regular toothpaste use maintains the white surface of the enamel by binding to plaque bacteria and not allowing plaque to accumulate as much.

Based on the evidence, we herein propose that HAP works in different ways to immediately whiten teeth, then with continued use, it provides long-lasting whiteness. Figure 2 shows how HAP works to achieve this.

#### 4.3.1. Immediate Whitening Effect

The use of HAP toothpaste provides an immediate whitening effect by providing micro-crystals of HAP that fill micro-defects on the surface of the enamel and remineralize the lost apatite from the surface [41], and providing adhesion to the enamel surface [46]. Moreover, gentle removal of plaque and stain results in whitening, which is achieved without heavy abrasion [44]. These effects are shown in Figure 2.

#### 4.3.2. Long-Term Whitening

With repeated daily use, and the interaction of HAP with saliva, HAP toothpaste eventually provides an opaque, white HAP deposit which partially, or completely, covers the enamel surface, as shown by the SEM results of Fabritius-Vilpoux et al. 2021 [64] and according to a study in this review [37]. This white layer not only makes the tooth appear whiter, but also blocks the reflection of incoming light off the darker yellow dentin back out to the observer. Moreover, HAP has been shown to bind to plaque bacteria and prevent their accumulation on the tooth surface [31]. The net result of the long-term use of HAP-containing toothpaste between HAP and the tooth surface is the safe improvement of the appearance of the teeth (see Figure 2).

### 4.4. Effects of Increasing Concentration of HAP in Toothpaste

The effects of HAP on the toothpaste are dose-dependent. Some studies have shown an increased effectiveness in dentin sensitivity with the increasing HAP content [65] and, in this review, we note that 0.25% was not very effective in tooth whitening, whereas there was a dose-response of whitening when 3% HAP was compared to 15% HAP [49]. There is other evidence that HAP deposition on the tooth surface is dose-dependent [64]. Therefore, a dose-response-relationship between HAP-concentration and both whitening effect and adhesion to the enamel is likely, as shown in these studies [49,64,65].

### 4.5. Limitations of the Review

The limitations of this review are as follows. While this review has unlikely omitted any study with evidence that HAP toothpastes whiten teeth, the in vivo evidence lacks high-quality RCTs of sufficient rigor, in order to make firm conclusions. For this reason, and due to the heterogeneity between the clinical trial conditions and oral care products used, a Cochrane-style meta-analysis could not be conducted. However, there is sufficient evidence from in vitro trials to conclude that HAP can whiten teeth, but this needs to be confirmed with more high-quality RCTs. The public demands oral care products that noticeably whiten teeth, which indicates that more clinical trials are needed.

### 4.6. The Future of Hydroxyapatite Toothpastes

Reviews of the published clinical evidence show that hydroxyapatite toothpastes assist in (a) reducing caries through remineralization and other mechanisms [28], (b) reducing dentin hypersensitivity by remineralizing the exposed dentin, defective enamel in molar incisor hypocalcification [31], or white spot enamel lesions [66], and (c) improving tooth color through whitening effects (Table 3). Therefore, hydroxyapatite is a versatile ingredient in oral care products, which provides multiple proven benefits.

## 5. Conclusions

This systematic review has shown a moderately high level of in vitro evidence, as well as some preliminary clinical (in vivo) evidence, that oral care products containing hydroxyapatite effectively and safely whiten teeth. This is achieved in the short-term, and with prolonged maintenance, with minimal abrasion, with plaque removal, by deposition of a white opaque layer on the surface of the enamel, and by repairing enamel defects without any oxidation of dental hard or soft tissue. High-quality RCTs, that extend and broaden the evidence presented herein, would assist in further establishing hydroxyapatite as an effective dental whitening agent in over-the-counter oral care products for home use.

## Figures and Tables

**Table 1:** Overview of the in vitro hydroxyapatite (HAP) studies in the field of tooth whitening (sorted by publication year, in descending order).

Study (Country) [Reference]	Condition	Tested Materials/Products	Experimental Conditions and Results	Quoted Conclusion of the Publication	% RoB Scores * Strength of the Finding of HAP Whitening Effect
Hojabri and Kunzelmann 2022 (Germany) [37]	in vitro	Mixture of HAP (6.25%) and P11-4 self-assembling peptide (Curodont Repair).	Fifty bovine polished incisors treated with bleach immersed for 30 s in test and control pastes; -color changes measured by spectrophotometry; -color change ∆E = 1.27 ± 1.32; -*p* = 0.007 in contrast to water control.	“Pre-treatment with a low-concentrated NaOCl enhanced the adherence of the HAP layer on the enamel surface, resulting in a stronger whitening Effect”.	79.2% HAP-whitening effect confirmed.
Shang et al. 2022 (Germany) [38]	in vitro	Toothpastes with 1% and 10% HAP.	Forty bovine incisor crowns polished, then stained in the lab; -toothpaste slurries applied with a toothbrush, then agitated, and color changes measured by spectrophotometry; - color change ∆E = 4.47; *p* < 0.05.	“nano-HAP toothpaste has a satisfying post brushing whitening effect and good resistance to mechanical forces. The whitening effect seemed to be concentration-dependent”.	79.2% HAP-whitening effect confirmed (10% HAP was more efficient than 1% HAP).
Shang and Kunzelmann 2021 (Germany) [39]	in vitro	HAP (3 µm, 200 nm, and 50 nm particle size), commercial whitening mouth rinse, distilled water.	Fifty bovine incisor crowns polished, then stained in the lab; -exposure simulated for 3–6 months of mouth rinsing; -color changes measured by spectrophotometry; -color change ∆E = 3.07; *p* < 0.01.	“The HAP nanoparticles showed better tooth-whitening performance after a longer period of mouthrinsing than the microsized HAP particles”.	79.2% HAP-whitening effect confirmed.
Hojabri et al. 2021 (Germany) [40]	in vitro	Experimental peptide-HAP suspensions (0.5% HAP, 6.25% HAP), commercial bleaching agent.	Forty bovine incisor crowns polished, then stained in the lab; -immersion in test mixtures for different times, then colorimetric color change; -color change ∆E = 6.42; -significant difference.	“The peptide-HAP suspension is a mild tooth whitener, and the adhesion of peptide-HAP to enamel is concentration dependent”.	79.2% HAP-whitening effect confirmed.
Sarembe et al. 2020 (Germany) [41]	in vitro	Fifteen percent of HAP gel (Karex Gelée), whitening mouth rinse with phosphates, distilled water.	Twenty-eight bovine cleaned incisors immersed in test paste/rinse of 9 cycles; -color changes measured by spectrophotometry; -color change ∆E = 11.2 [±3.11]; *p* < 0.0001.	“This in vitro study demonstrated a significantly higher ad hoc whitening effect of the HAP gel compared to the mouth rinse and water after short-time application”.	75% HAP-whitening effect confirmed.
Bommer et al. 2018 (Switzerland, Germany) [42]	in vitro	Mixture of self-assembling peptide matrix and 12.5% HAP.	Twenty stained bovine incisors; -micro brush application of mixture for 5 min, rinsed, and color changes measured by a colorimeter; -color change ∆E = 4.8 [±3.6]; *p* < 0.002.	“The combination of SAPM+HA particles caused optical whitening based on diffuse reflection by the HA particles on the tooth surface. The whitening effect and its magnitude observed in vitro were also seen *in vivo*”.	71% HAP-whitening effect confirmed.
Jin et al. 2013 (Germany) [43]	in vitro	Toothpastes containing 10%, 20%, and 30% HAP; controls: A commercial toothpaste, a topical fluoride agent.	Ninety extracted caries-free polished human teeth; -3 min of application using a cotton pellet, allowed to sit for 5 min, agitated or stored, and color changes measured by a colorimeter; -color change ∆E = 0.91 [±0.50]; *p* < 0.05.	“Calcium phosphate-based formulations that can adhere to the enamel surface and contribute to tooth whitening have promising tooth-whitening potential”.	79.2% HAP-whitening effect confirmed (30% Zn-HAP more efficient than both 20% Zn-HAP and 10% Zn-HAP).
Kim et al. 2011 (South Korea) [44]	in vitro	Ten percent of nano-carbonate apatite, casein phosphopeptide-amorphous calcium phosphate, NaF, distilled and deionized water.	Twenty-four bovine bleached incisors; -3-min treatments, four times per day, with test and control pastes, then pH cycled in artificial saliva to remineralize; -color change ∆E = 5.26 [±2.28]; *p* < 0.05.	“10% nano-carbonate apatite could significantly maintain the initial color and protect the damaged enamel structure after bleaching”.	79.2% HAP-whitening effect confirmed.
Dabanoglu et al. 2009 (Germany) [45]	in vitro	Three HAP suspensions and two HAP mixtures in dissolvable polymer films.	Thirty extracted human premolars; -applied suspensions for 3 min with a cotton pellet and agitation, allowed to sit for 5 min, then rinsed; -color changes were measured by spectrophotometry.	“the materials used in the study are very promising alternatives to oxidizing bleaching agents”.	79.2% HAP-whitening effect confirmed.
Park et al. 2007 (South Korea) [46]	in vitro	Toothpaste with 15% sodium metaphosphate, toothpaste with 15% nano-HAP.	Sixty sectioned bovine incisors imbedded in resin and mechanically brushed 20,000 strokes (50 strokes/min) with the toothpastes; -a shade guide was used to judge whiteness.	“with the one (dentifrice) with nano-hydroxyapatite, (whitening) is achieved by the adhesion to organic substance on enamel surface”.	71% HAP-whitening effect confirmed.
Kim et al. 2006 (South Korea) [47]	in vitro	Newly developed toothpaste containing nano-sized HAP and two commercial toothpastes (one containing silica and multi-phosphate and the other containing silica and micro-sized HAP).	Sixty-six human extracted human molar crowns imbedded in resin and mechanically brushed 20,000 strokes (50 strokes/min) with the toothpastes; -a shade guide was used to judge whiteness.	“they (new nano-HA toothpaste) had a similar whitening efficacy to commercially available whitening toothpastes”.	75% HAP-whitening effect confirmed.
Park et al. 2006 (South Korea) [48]	in vitro	Four types of toothpaste slurries including 40% calcium carbonate, 15% dicalcium phosphate, 15% sodium metaphosphate, and 12% hydrated silica + 3% nano-HAP.	Eighty natural human teeth imbedded in resin and mechanically brushed 20,000 strokes (50 strokes/min) with the toothpastes; -a shade guide was used to judge whiteness.	“Unlike the result that sodium metaphosphate-included dentifrice has whitened the teeth through the abrasion of the hard tissue, it is judged that the nano-hydroxyapatite have been attached to the hard demineralized tissue and improving the teeth-whitening”.	75% HAP-whitening effect confirmed.
Niwa et al. 2001 (Japan) [49]	in vitro	Toothpastes containing 0%, 3%, and 15% HAP.	Five artificial sintered hydroxyapatite tooth blocks were tested for polishing effects of different HAP concentrations; -thickness loss (nm/cm^3^/h) was measured with each HAP paste.	“Adding different amounts of hydroxyapatite to toothpaste does not change the polishing properties”.	66.7% (15% HAP was no different in polishing efficiency than 3% HAP); -statistics were inadequate.

* The % scores were based on the QUIN scores by Sheth 2022 [35]: >70% = low risk of bias, 50% to 70% = medium risk of bias, and <50% = high risk of bias using the following formula: Final score = Total score × (100)/(2 × number of criteria applicable).

**Table 2:** QUIN Tool scores [35] of in vitro whitening studies. Scores for studies are awarded according to the following. Adequately specified = 2; inadequately specified = 1; not specified = 0; not applicable indicates that this category would not be counted.

Study [Ref.]	Clearly Stated Aims, Objectives	Detailed Explanation of Sample Size Calculation	Detailed Explanation of Sampling Technique	Details of Comparison Group	Detailed Explanation of Methodology	Operator Details	Randomization	Method of Measurement of Outcome	Outcome Assessor Details	Blinding	Statistical Analysis	Presentation of Result	Score	%
Hojabri 2022 [37]	2	0	2	2	2	2	1	2	2	0	2	2	19 (100)/24	79.1
Shang 2022 [28]	2	0	2	2	2	2	1	2	2	0	2	2	19 (100)/24	79.1
Shang 2021 [39]	2	0	2	2	2	2	1	2	2	0	2	2	19 (100)/24	79.1
Hojabri 2021 [40]	2	0	2	2	2	2	1	2	2	0	2	2	19 (100)/24	79.1
Sarembe 2020 [41]	2	0	2	2	2	2	0	2	2	0	2	2	18(100)/24	75
Bommer 2018 [42]	2	0	2	1	2	2	0	2	2	0	2	2	17(100)/24	71
Jin 2013 [43]	2	0	2	2	2	2	1	2	2	0	2	2	19 (100)/24	79.1
Kim 2011 [44]	2	0	2	2	2	2	1	2	2	0	2	2	19 (100)/24	79.1
Dabanoglu 2009 [45]	2	0	2	2	2	2	1	2	2	0	2	2	19 (100)/24	79.1
Park 2007 [46]	2	0	2	1	2	2	0	2	2	0	2	2	17 (100)/24	71
Kim 2006 [47]	2	0	2	2	2	2	0	2	2	0	2	2	18 (100)/24	75
Park 2006 [48]	2	0	2	2	2	2	0	2	2	0	2	2	18 (100)/24	75
Niwa 2001 [49]	2	0	2	2	2	2	0	2	2	0	1	1	16 (100)/24	66.7

## Data Availability

Not applicable.

## Supplementary Materials

The following supporting information can be downloaded at: https://www.mdpi.com/article/10.3390/dj11020050/s1. PRISMA-S checklist [67].
